# Ultrastructure of book gill development in embryos and first instars of the horseshoe crab *Limulus polyphemus *L. (Chelicerata, Xiphosura)

**DOI:** 10.1186/1742-9994-9-4

**Published:** 2012-03-21

**Authors:** Roger D Farley

**Affiliations:** 1 Department of Biology, University of California, Riverside, CA 92521, USA

**Keywords:** Horseshoe crab, *Limulus polyphemus*, Book gills, Endoskeleton, Cartilage, Development, Embryos, First instar, Book lungs, Cuticle

## Abstract

**Background:**

The transmission electron microscope (TEM) is used for the first time to study the development of book gills in the horseshoe crab. Near the end of the nineteenth century the hypothesis was presented for homology and a common ancestry for horseshoe crab book gills and arachnid book lungs. The present developmental study and the author's recent ones of book gills (SEM) and scorpion book lungs (TEM) are intended to clarify early histological work and provide new ultrastructural details for further research and for hypotheses about evolutionary history and relationships.

**Results:**

The observations herein are in agreement with earlier reports that the book gill lamellae are formed by proliferation and evagination of epithelial cells posterior to opisthosomal branchial appendages. A cartilage-like endoskeleton is produced in the base of the opisthosomal appendages. The lamellar precursor cells in the appendage base proliferate, migrate outward and secrete the lamellar cuticle from their apical surface. A series of external, posteriorly-directed lamellae is formed, with each lamella having a central channel for hemolymph and pillar-type space holders formed from cells of the opposed walls. This repeated, page-like pattern results also in water channels (without space holders) between the sac-like hemolymph lamellae.

**Conclusions:**

The developmental observations herein and in an earlier study (TEM) of scorpion book lungs show that the lamellae in book gills and book lungs result from some similar activities and features of the precursor epithelial cells: proliferation, migration, alignment and apical/basal polarity with secretion of cuticle from the apical surface and the basal surface in contact with hemolymph. These cellular similarities and the resulting book-like structure suggest a common ancestry, but there are also substantial developmental differences in producing these organs for gas exchange in the different environments, aqueous and terrestrial. For scorpion book lungs, the invaginated precursor cells align in rows and secrete rows of cell fragments that are the basis for the internal, anterior-directed air sacs. The hemolymph sacs of book gills are formed by epithelial evagination or outfolding from the posterior surface of the branchial appendages.

## Introduction

As reviewed by Farley [1,2], the page-like organization of horseshoe crab book gills and arachnid book lungs has suggested homology and a common ancestry, and the structural similarity of arachnid book lungs has raised the possibility of a common terrestrial origin [3]. Numerous earlier workers used light microscopy to compare book gill and book lung development [4-21].

In the present investigation of book gill development in the American horseshoe crab, the light microscope (LM) and scanning (SEM) and transmission electron microscopes (TEM) are used to extend an earlier investigation with SEM [1]. The latter study provides an overview of the opisthosomal appendages and book gills as they enlarge in the embryo and first and second instars. Fractured tissues examined with the SEM show the appendage and book gill cuticle is underlain by an epithelial layer, the hypodermis, that secretes the cuticle. This supports the earlier histological observations [11] that the gill lamellae are an epithelial evagination or outgrowth from the posterior surface of the opisthosomal branchial appendages.

The semi-thin and ultrathin sections of the present study provide a more detailed view of the cellular formation of the opisthosomal appendages and gill lamellae and their pillar-type space holders. Results are compared with the ultrastructure of scorpion book lung development [2]. The objective is to use modern microscopy to clarify and extend the early histological studies and provide additional developmental details for hypotheses about evolutionary history and for further investigations such as those for gene expression.

The early horseshoe crab embryos develop within an outer egg envelope (chorion) and an inner egg membrane (deutovum). The outer envelope is eventually ruptured, and the remaining inner membrane expands as the embryo increases in size. The embryos undergo four molts before hatching 13-33 days after fertilization [1,22-26].

The present investigation begins after the second embryonic molt (stage 19) [25,26] before opisthosomal appendages have started to develop. After the third embryonic molt (stage 20), the genital operculum and first branchial appendages are flap-like lobes, but they are immobile. The prosomal appendages of these post third-molt embryos are active, and the entire embryo moves about inside its membranous covering [1,25,26].

During the 1-2 weeks before hatching, the post fourth-molt embryo (stage 21) is very active inside its covering, and now there is often rhythmic beating of the operculum and first branchial appendage, the latter with some attached gill lamellae [1,25,26].

The post fourth-molt embryos emerge as free-swimming first instars (trilobite larvae). Hatching is increased with agitation, hypoosmotic shock and other conditions likely to occur during periods of high water [27,28]. This is thought to increase chances the larvae will survive in the water column rather than perish on an exposed beach. The first instars do not feed, but are very active, relying on stored maternal nutrients [1].

Depending on conditions, the first instars molt to second instars in 9-15 days [26], and the latter begin feeding. The second instars have prominent first and second branchial appendages, and gill lamellae extend posteriorly from the posterior surface of each bilateral lobe of these appendages [1]. The lobes of the third branchial appendages are barely evident on the opisthosomal ventrum.

In the present investigation, opisthosomal appendage and gill lamellar development is examined in stages 19-21 after the second, third and fourth embryonic molts and in first instars. The use of TEM provides more developmental details and extends earlier observations [1,2] that there are important similarities and differences in the cellular activities that produce the page-like lamellae of book gills and scorpion book lungs.

## Results

### Early development of opisthosomal appendages and gill lamellae

**Figure 1:**
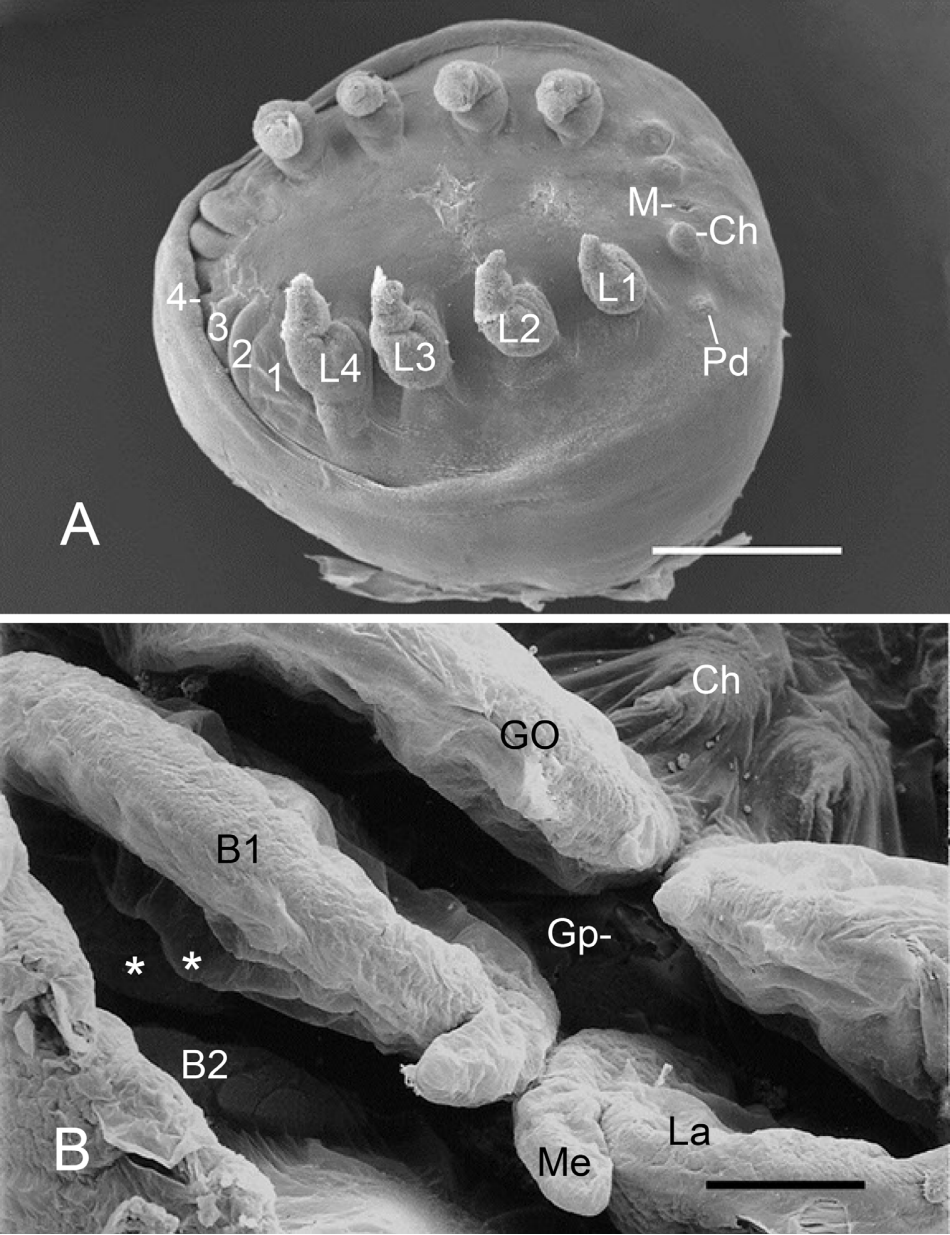
**Ventral view of embryos of the horseshoe crab. *Limulus polyphemus*, supine. SEMs**. **A**. After the second embryonic molt (stage 19), the prosomal legs (L1-L4) are prominent, tapered and starting to become segmented, while the opisthosoma only has bilateral ridges in each segment (1-4). The development of the chelicerae (Ch) and pedipalps (Pd) are unusually delayed in this specimen. M, mouth. Scale, 500 μm. **B**. After the third embryonic molt (stage 20), the first opisthosomal segment has small bilateral lobes, the future chilaria (Ch). In the following three opisthosomal segments, the genital operculum (GO) and first (B1) and second (B2) branchial appendages are bilateral flap-like structures with small median (Me) and large lateral (La) lobes. These appendages are all immobile at this stage. Barely discernible are primordial gill lamellae (asterisks) from the posterior surface of the first branchial appendage. Gp, gonopore. Scale, 120 μm.

As shown in Farley [1] and earlier publications [25,26], the appendages of the opisthosoma usually develop more slowly than those in the prosoma. The pedipalps and chelicerae are most likely to be prominent after the first (stage 18) and second (stage 19) embryonic molts [1,25,26], but there are developmental variations as in the post-second molt embryo of Figure 1A where the pedipalps and chelicerae are delayed. The prosomal legs at this stage are tapered and starting to become segmented, while in the opisthosoma there are bilateral ridges in the four opisthosomal segments of the future chilaria, genital operculum and the first and second branchial appendages.

After the third molt (stage 20; Figure 1B), there are still small lobes for the future chilaria, but the genital operculum and first branchial appendages are now bilateral flaps, each with a small median and large lateral lobe. Small bilateral ridges posterior to the first branchial appendage are the beginnings of the second branchial appendage (Figure 1B) [1].

**Figure 2:**
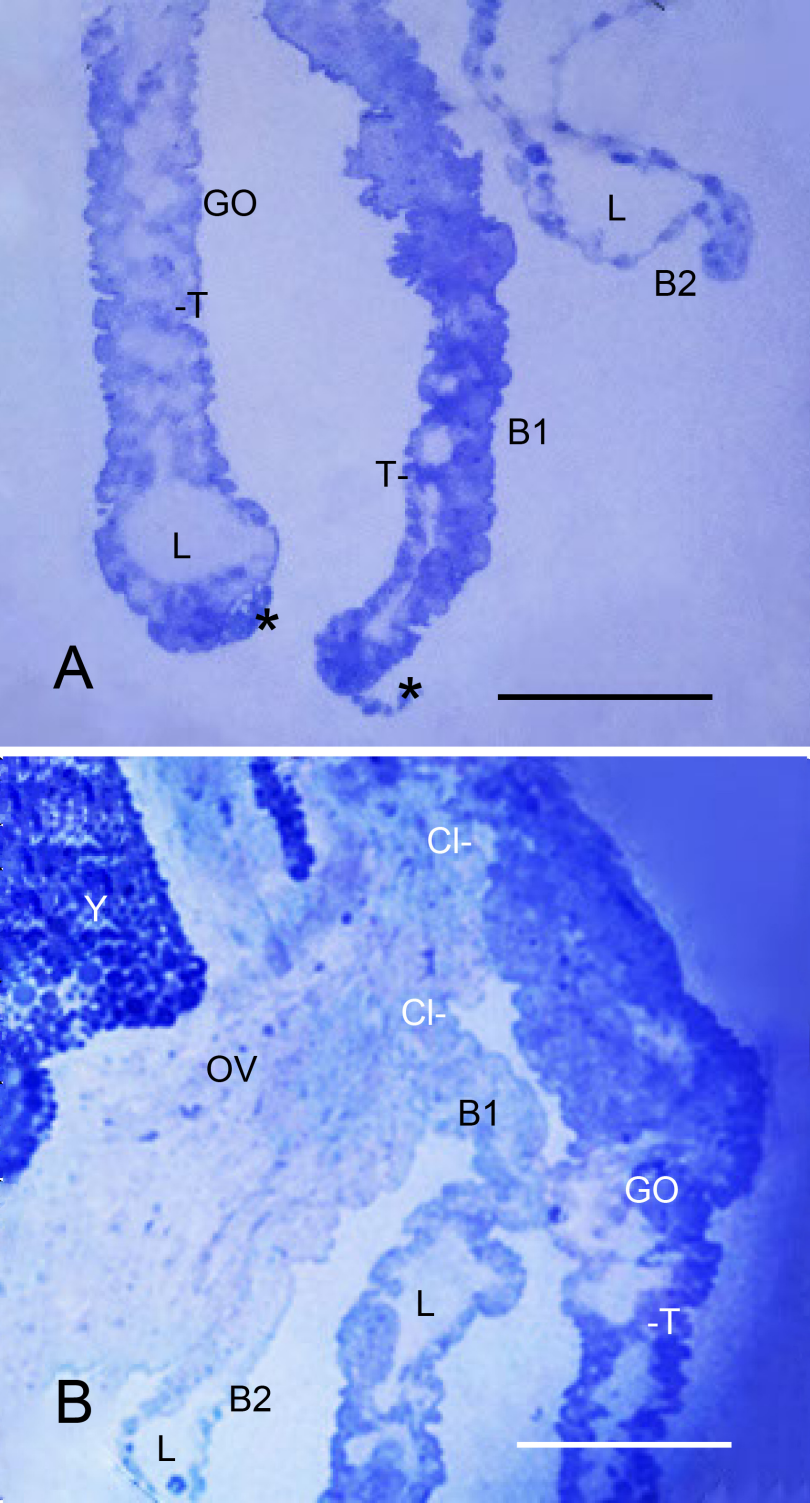
**Thin distal and thick proximal parts of the genital operculum (GO) and first branchial appendage (B1) after the third embryonic molt (stage 20)**. *Limulus polyphemus*, prone. Semi-thin sections, LMs. Sections through these flap-like appendages (Figure 1B) show that the initial distal parts have a knobby appearance at this stage as a result of the outward protrusion of cells of the outer hypodermal layer (Figures 3, 4). **A**. A central lumen (L) is present in some places in the thin distal parts of the appendages shown here, including the second branchial appendage (B2). As shown in more detail below, the outer wall of the appendages consists of a layer of hypodermal cells and the cuticle they secrete. At intervals along the length of the appendages, some hypodermal cells extend processes into the lumen to start forming pillar-type trabeculae (T). Also at this stage, as further described below, a small cluster of cuticular vesicles (asterisks) is evident at the tips of the genital operculum and first branchial appendage. Scale, 115 μm. **B**. A narrow cleft (Cl) or space is evident between the thick proximal bases of the genital operculum and first branchial appendage as they lengthen from the opisthosomal ventrum (OV). The thin distal regions evident here have a central lumen (L) and developing trabeculae (T). B2, distal part of the second branchial appendage; Y, yolk. Scale, 120 μm.

**Figure 3:**
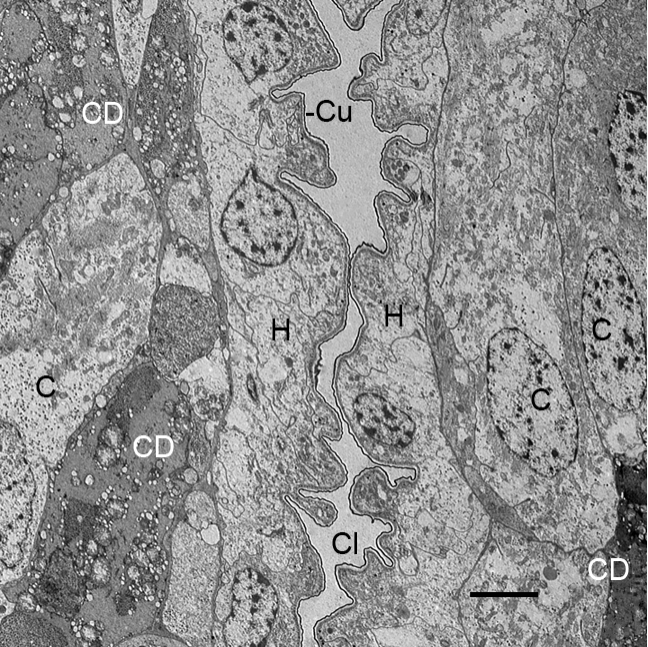
**Narrow cleft (Cl) like that in Figure 2B between the emerging thick proximal bases of the genital operculum (left) and first branchial appendage (right) after the third molt (stage 20)**. *Limulus polyphemus*, prone. TEM. Both appendages have an outer hypodermal layer (H) with only a very thin cuticle (Cu) at this stage. The central part of each appendage is filled with cells (C) and cell debris (CD), the latter apparently from cells that deteriorate in forming a central lumen for passage of hemolymph. Small hypodermal cell protrusions into the cleft give these appendages a knobby appearance that is typical of this stage (Figures 2A, B and 4). The external, ventral surface of the embryo is toward the bottom of the photo. Scale, 5 μm.

Just after the third embryonic molt, the flap-like genital operculum and branchial appendages consist of a thin distal region (Figures 1B, 2A, B) while the thicker proximal bases of these appendages are just starting to extend from ventral surface of the opisthosoma. In Figure 2B, there is a narrow cleft or space between the developing proximal parts of the genital operculum and first branchial appendage. The electron micrograph in Figure 3 shows the cleft and the adjacent tissue of these appendages. At this stage, both appendages have a hypodermal layer and thin external cuticle. Farther inward in both appendages, the cell debris suggests cell deterioration and formation of a central lumen for passage of hemolymph as evident in later stages.

**Figure 4:**
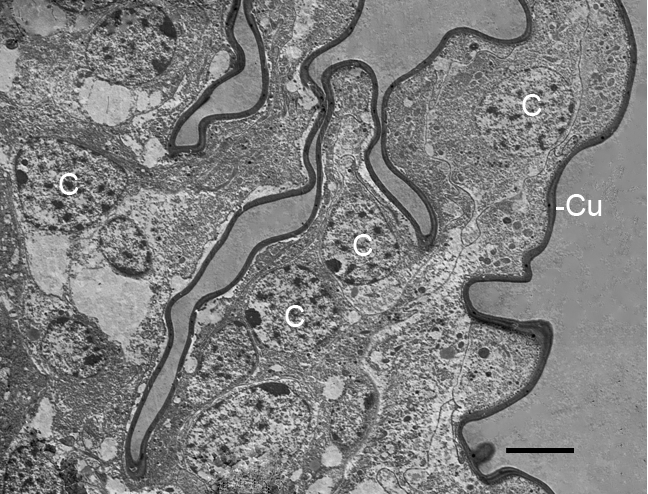
**A complex outgrowth of cells (C) from the base of the genital operculum after the third embryonic molt (stage 20)**. *Limulus polyphemus*. TEM. Such somewhat erratic outgrowths may be helpful for gas exchange, and they result in a knobby outer wall as commonly occurs for opisthosomal appendages at this stage (Figures 2A, B and 3). As described in more detail below, a thin cuticle (Cu) is secreted by these cells. Scale, 5 μm.

After the third embryonic molt, sections through the genital operculum, first and second branchial appendages and later gill lamellae show these structures have a knobby appearance due to the hypodermal cells evaginating outward at the tip and along the length of these appendages and lamellae (Figures 2A, B and 3). This increases surface area for gas exchange and is probably indicative of the growth process, resulting in increased length and width of these structures. The knobby appearance is a prominent feature of post third-molt (stage 20) embryos but much less so of later stages. The outward cellular growth is sometimes quite elaborate, especially near the base of the appendages. Figure 4 shows cells extending processes from the base of the genital operculum, and as described below, these cellular outgrowths produce a cuticular covering along the way. Gill lamellae do not emerge from the genital operculum, so the knobby outgrowths like those in Figures 2A, B, 3 and 4 apparently increase the gas exchange surface and increase appendage size rather than form a distinctive new structure.

**Figure 5:**
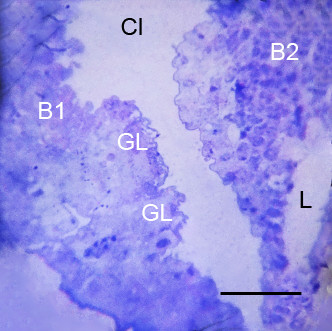
**Two gill lamellae (GL) starting to develop from the posterior surface of the thick, proximal base of the first branchial appendage (B1) after the third embryonic molt (stage 20)**. *Limulus polyphemus*, prone. Semi-thin section, LM. The outward growth of the hypodermal cells gives these appendages a knobby appearance as described above (Figures 2A, B and 3, 4). The gill lamellae at this early stage of development are not yet organized into an external hypodermis and central lumen (Figures 6, 7). A narrow cleft (Cl) is evident between the bases of the first and second (B2) branchial appendages. L, lumen. Scale, 45 μm.

**Figure 6:**
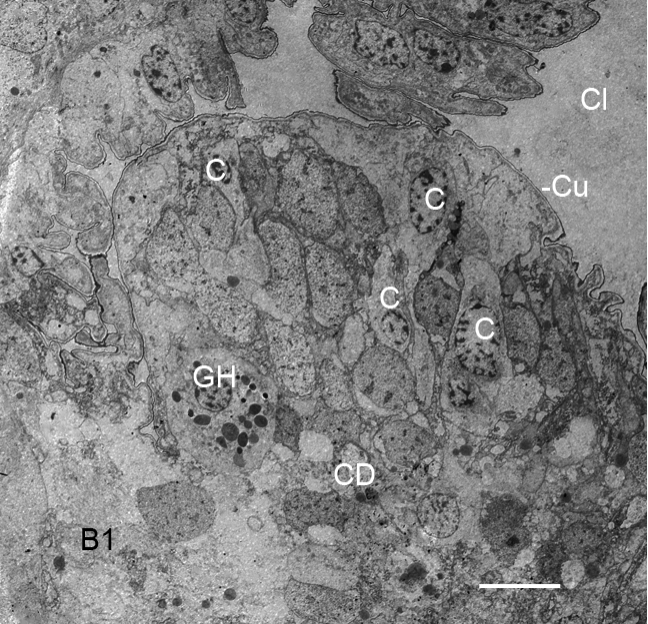
**Early gill lamellae starting to develop from the posterior surface of the first branchial appendage (B1) after the third embryonic molt (stage 20)**. *Limulus polyphemus*, prone. TEM. The lamella at this early stage of development is not yet organized into an outer hypodermis and central lumen as evident later in Figure 7. Some peripheral cells (C) appear to be intact as though they could become part of a hypodermal layer. Cell debris (CD) at the center and base of the lamella suggests the beginning formation of a lumen. The lamella has a very thin external cuticle (Cu) at this stage. Cl, narrow cleft (Figures 2B, 3, 5) between the bases of the first and second branchial appendages; GH, granular hemocyte [29-31]. Scale, 10 μm.

As the thick proximal part of the first branchial appendage extends from the opisthosomal ventrum, small clusters of cells may be seen protruding outward from the posterior surface of this part of the appendage (Figure 5). In the SEM view in Figure 1B, small lobes (indicated by asterisks) are probably early gill lamellae emerging from the posterior surface of the first branchial appendage. Initially, the primordial gill lamellae may appear as a small outfolding of a double layer of hypodermis and cuticle. More commonly the early lamella is a somewhat disorganized group of cells (Figures 5, 6), with those at the periphery apparently intact while there is cell debris near the center (Figure 6). Cells with large (~1 μm dia.) granules are probably hemocytes [29-31] carried into the area with the hemolymph. Patten and Hazen [32] suggest that opisthosomal somites are a source of at least some hemocytes.

**Figure 7:**
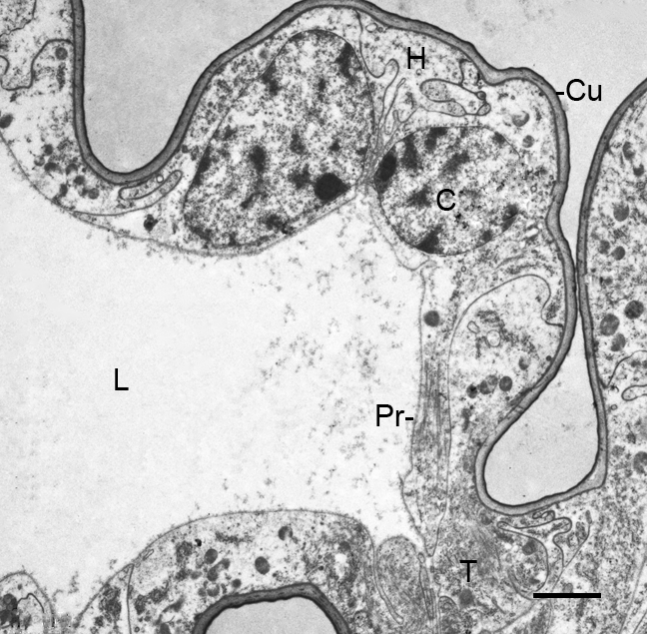
**A region of a gill lamella after the fourth embryonic molt (stage 21)**. *Limulus polyphemus*, prone. TEM. This lamella is at a more advanced stage than those in Figures 5 and 6, and it has a clear central lumen (L), an outer hypodermal layer (H) and a thin cuticle (Cu). At this site, two cells protrude outward as commonly occurs among the appendages and lamellae at this early stage (Figures 2A, B and 3, 4, 5, 6). One hypodermal cell (C) is extending a process (Pr) toward a developing pillar-type trabecula (T; Figures 2A, B). The fine particulate material in the lumen is probably hemocyanin [30,31]. Scale, 2 μm.

Although initially somewhat variable, the early gill lamellae soon have a structure like that of the genital operculum and branchial appendages: a knobby outer layer of hypodermal cells and external cuticle (Figures 2A, B and 3, 4, 5, 6, 7) and a lumen presumably filled with hemolymph (Figures 2A, B and 7). At intervals along the length of the appendages and gill lamellae, hypodermal cells from opposed lamellar walls extend processes inward to start forming pillar-type space holders (Figures 2A, B and 7).

**Figure 8:**
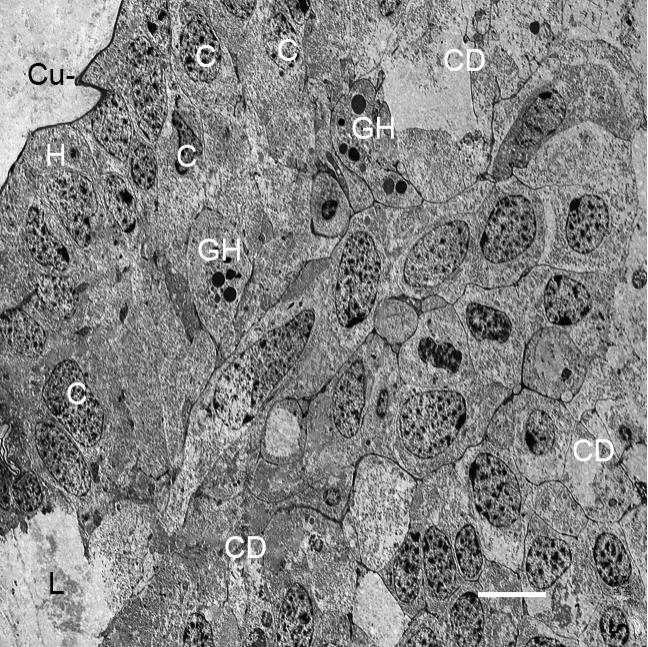
**Diversity of cells and cell debris in the thicker, proximal base of the genital operculum after the third embryonic molt (stage 20)**. *Limulus polyphemus*, prone. TEM. Some cells (C) near the periphery appear to be intact as though they could become part of the developing hypodermis (H). Other cells are deteriorating, leaving cell debris (CD), and thus making more space for the central lumen (L) and passage of hemolymph. A thin external cuticle (Cu) is evident. GH, granular hemocytes [29-31]. Scale, 5 μm.

Initially, the thick, proximal bases of the genital operculum and branchial appendages are filled with cells, and a lumen is only starting to form (Figures 2B, 3, 5). The electron micrograph of Figure 8 shows some of the many cells at the base of a genital operculum after the third embryonic molt (stage 20). Cells near the center of the operculum base are deteriorating as though forming the appendage lumen while others near the periphery appear to be intact as a hypodermal layer. Interspersed among the other cells, those with large (~1 μm dia.) granules are probably hemocytes [29-31] carried into the area with the hemolymph.

**Figure 9:**
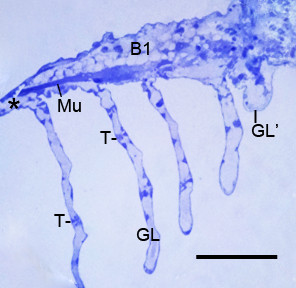
**The thick proximal base of the first branchial appendage (B1) after the fourth embryonic molt (stage 21)**. *Limulus polyphemus*, prone. Semi-thin section, LM. The thin distal end (*) of the appendage extends at left beyond the micrograph. This proximal base of the appendage is the source of the gill lamellae (GL). Their decreasing length toward the proximal end of the appendage (right) indicates their sequence of formation. The space between the lamellae is variable; an early gill lamella (GL') is very close to the preceding lamella. The central region of the appendage base has a band of muscle cells (Mu) and large vacuoles, probably from deteriorating cells. The gill lamellae have a thin outer wall of hypodermis and cuticle (Figure 7), and pillar-type trabeculae (T) are starting to form at intervals along the length of the lamellae. Scale, 130 μm.

The gill lamellae do not have distinctive distal and proximal parts as occurs in the opisthosomal appendages (Figures 2A, B and 3, 5, 8), and the gill lamellar lumen is a prominent feature very early in their development (Figures 7, 9). After the fourth embryonic molt, the lumina of appendages and gill lamellae often contain cell debris, hemocytes and fine particulate material that is most likely hemocyanin (Figure 7) [30,31].

Figure 9 shows the proximal base of the first branchial appendage after the fourth embryonic molt when this region is fully extended from the ventral surface of the opisthosoma. There is variable spacing between the gill lamellae, but the distance tends to decrease toward the proximal base of the appendage. The gill lamellae also have decreased length toward the appendage base, indicating their sequence of emergence from the posterior surface of the appendage (Figures 5, 9) [1]. In this basal part of the first branchial appendage in Figure 9, the central core has a band of muscle cells and large vacuoles that are the likely remains of deteriorated cells. Cell debris and cells in various stages of deterioration (swollen, disrupted) are common in the central core of the appendage bases (e.g., Figures 3, 8). The embryos contain much yolk material, and large masses of yolk-filled cells are evident during dissection of the opisthosoma and in light and electron micrographs of this region (e.g., Figure 2B).

### Pillar-type trabeculae

**Figure 10:**
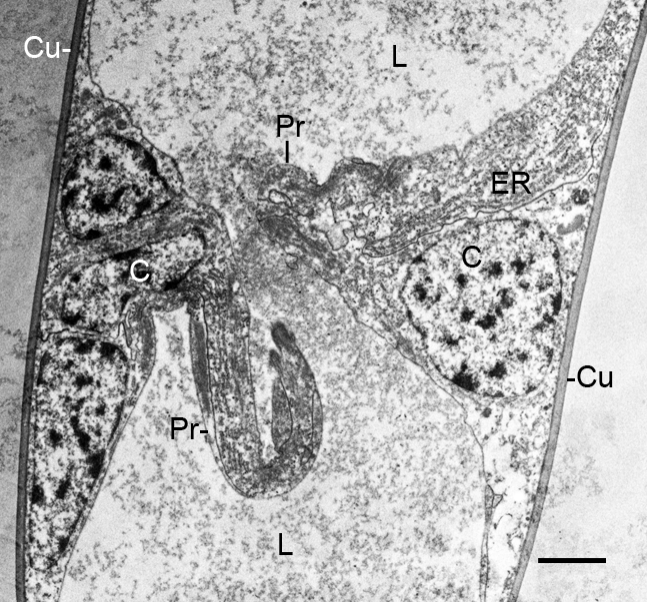
**Developing pillar-type trabecula in the thin distal part of the genital operculum after the fourth embryonic molt (stage 21)**. *Limulus polyphemus*. TEM. Hypodermal cells (C) from the opposed walls of the appendage extend processes (Pr) into the lumen (L) where the ends of the cell processes have not yet formed a connection. The lumen contains fine particulate material that is probably hemocyanin [30,31]. Cu, cuticle; ER, endoplasmic reticulum. Scale, 2 μm.

**Figure 11:**
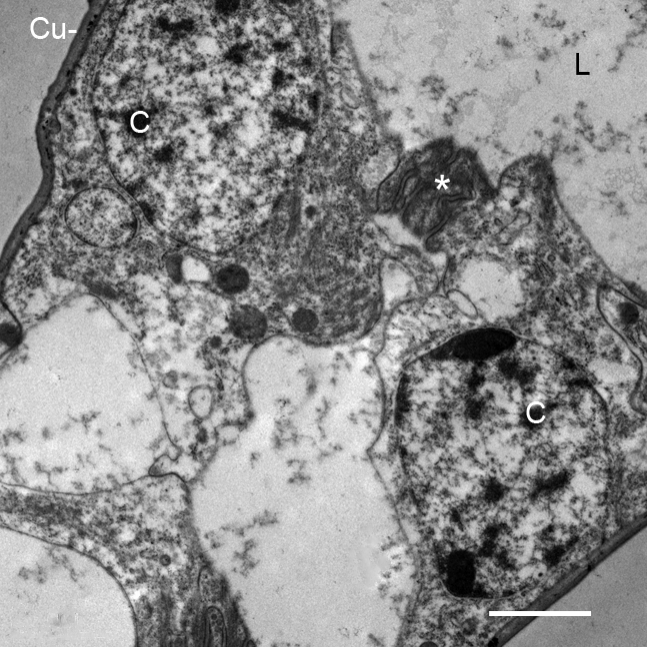
**Pillar-type trabecula in the thin distal part of the genital operculum after the fourth embryonic molt (stage 21)**. *Limulus polyphemus*. TEM. Hypodermal cells (C) from the opposed walls of the appendage extend processes that join in forming a complex connection (*). The lumen (L) has fine particulate material that is probably hemocyanin [30,31]. Cu, cuticle. Scale, 2 μm.

In post third-molt embryos, pillar-type space holders [33,34] are starting to form between cells from opposed walls of the genital operculum, first branchial appendage and the early gill lamellae (Figures 2A, B and 7, 9). Near the tips of the growing appendages, cells from the opposed walls extend processes toward each other and start the process of forming a trabecular pillar. Joining of opposed cells also occurs among those near the base of the appendages and at intervals along the length of the appendages (Figures 2A, B and 10, 11) and gill lamellae (Figures 7, 9). The cell processes from the opposed cells may not yet have made contact in these early stages (Figure 10), while others appear to be forming a complex connection (Figure 11). In Figures 10 and 11, the fine particulate material in the appendage lumen is probably hemocyanin [30,31].

In the initial formation of trabeculae in opisthosomal appendages and gill lamellae, there may be some intercellular communication so that a cell on one wall can induce a response from a cell at the opposite wall. A common feature at this stage is a prominent cell process extending inward from one wall while the opposed wall has only a small lobe extending inward. The trabeculae become more numerous and more developed in later stages.

### Endoskeleton

**Figure 12:**
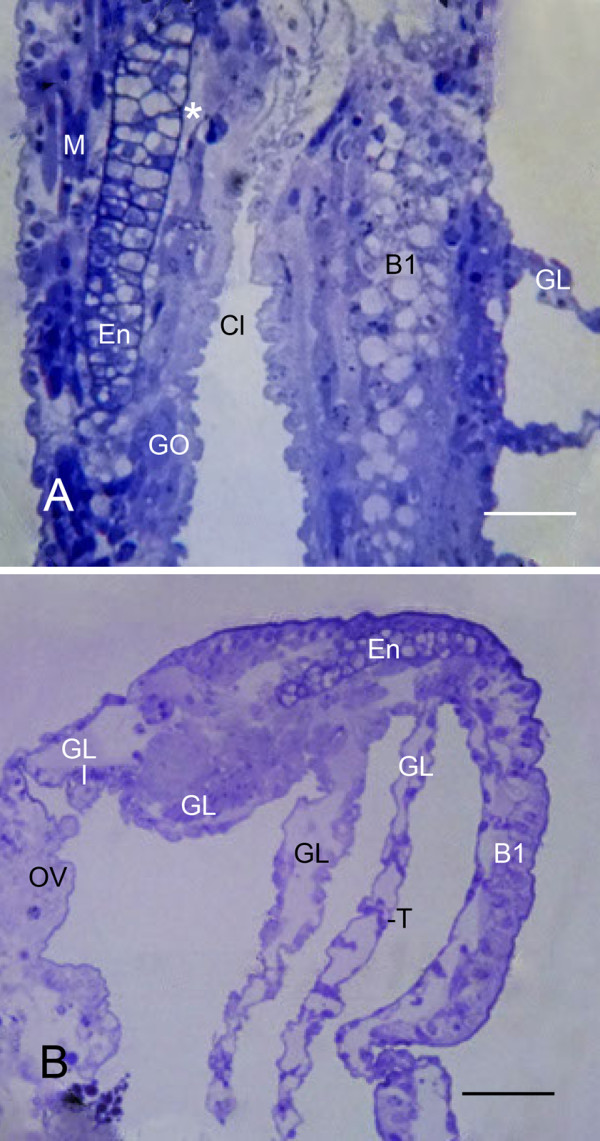
**Cartilage-like endoskeleton (En) in the thick, proximal base of opisthosomal appendages**. *Limulus polyphemus, *prone. Semi-thin sections, LM. **A**: In this example after the third embryonic molt (stage 20), the endoskeleton at the base of the genital operculum (GO) has a rod-like structure with cells surrounded by a thin, dense matrix. In the proximal end of the endoskeleton, some matrix chambers are swollen and without cell contents (*). The base of the first branchial appendage (B1) has large vacuoles, probably from deteriorated cells. Cl, cleft between the appendage bases; GL, gill lamella; M, muscle cells. Scale, 50 μm. **B**: Some endoskeleton is present at the thick base of the first branchial appendage (B1) in this first instar. The gill lamellae (GL) have variable spacing and decreasing development and length toward the proximal end (left) of the appendage. OV, opisthosomal ventrum; T, developing pillar-type trabeculae. Scale, 80 μm.

**Figure 13:**
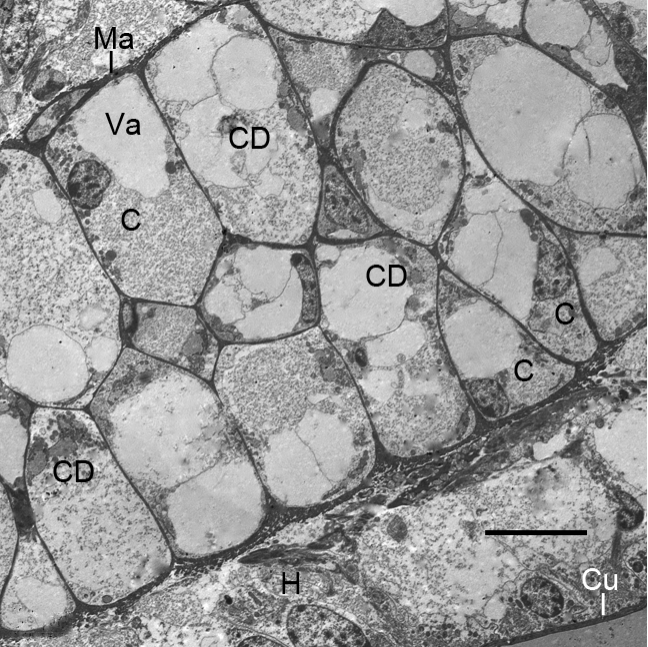
**Part of the cartilage-like endoskeleton in the thick proximal part of the genital operculum after the fourth embryonic molt (stage 21)**. *Limulus polyphemus*. TEM. Some cells (C) with nuclei have large vacuoles (Va), and cell debris (CD) is common. A thin, dense matrix (Ma) surounds each cell and the entire endoskeleton. Cu, external cuticle; H, hypodermal layer. Scale, 10 μm.

As described above for the post third-molt (stage 20) embryo, the genital operculum and first branchial appendages have a bilateral flap-like structure (Figure 1B), but they do not yet have mobility. As the thicker proximal bases of the genital operculum and first branchial appendage increase in length and extend from the surface of the opisthosomal ventrum (Figures 2B, 3, 5, 9), an endoskeletal structure may be included (Figure 12A, B). The endoskeleton consists of a rod-like mass of cells with each cell and the entire endoskeleton surrounded by a thin, dense matrix that most likely gives strength to the structure. The endoskeletal chambers may be swollen and without cell contents (asterisk in Figure 12A). The enclosed cells commonly have large vacuoles, and some matrix chambers only have cell debris (Figure 13). The cells and matrix chambers have diverse shapes, but are often somewhat rectangular, as much as 12 × 25 μm. The endoskeleton has some stability during dissection as shown in semi-thin sections through this region. A portion of the endoskeleton sometimes remains as the surrounding more delicate tissue is removed.

After the third and fourth embryonic molts, and in first and second instars, some endoskeleton was only seen in the basal region of the genital operculum (Figure 12A) and sometimes in the first branchial appendage (Figure 12B). In Figures 9 and 12A, the central core of the base of the first branchial appendage has large vacuoles from deteriorating cells, and there is as yet no endoskeleton. Reportedly, the endoskeleton is later in developing in the first branchial appendage as compared with the genital operculum [32]. The observed lengths of the endoskeletal shafts vary in the different examples seen herein, and an endoskeleton is more likely to be present in the genital operculum than in the first branchial appendage.

The endoskeleton resembles cartilage thought to be present in many invertebrates [31,32,35-39]. It apparently gives support to the basal portion of at least some of the embryonic appendages in their early stages of development. In the stages examined herein, an endoskeleton was not observed in the second branchial appendage or within the gill lamellae (Figures 5, 6, 7, 9, 12A, B).

### Cuticle formation

The process of cuticle formation is uncertain in these horseshoe crab embryos, but examination of tissues from post third-molt embryos gives some indication of how the cuticular layer is formed outside the epithelial cells as they extend processes at the tip and along the length of the developing appendages and gill lamellae.

**Figure 14:**
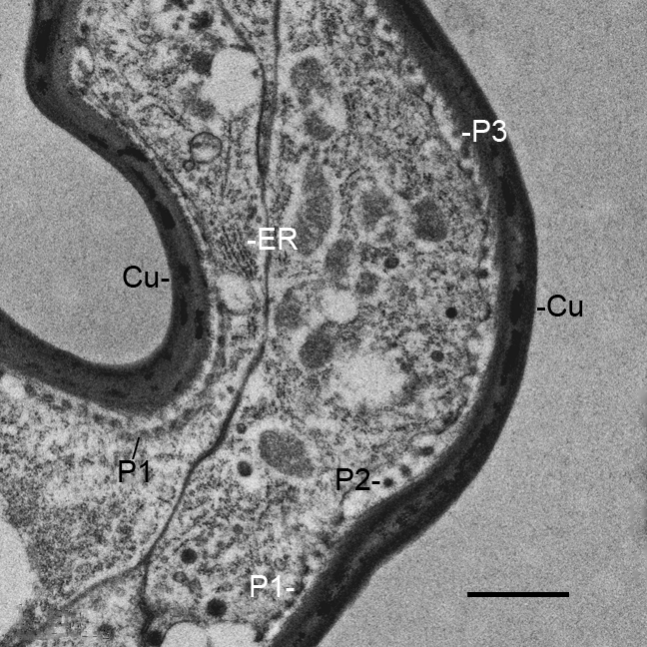
**Formation of external cuticle in an outgrowth of hypodermal cells (Figure 4) from the base of the genital operculum after the third embryonic molt (stage 20)**. *Limulus polyphemus*. TEM. At this place in the cellular outgrowth, the cell membrane is somewhat pulled away from the inner surface of the external cuticle (Cu). Small electron-opaque particles (P1) can be seen in a row attached to the cell membrane while some particles (P2) are in the space between the cell membrane and inner cuticle surface. Other particles (P3) are attached to the inner cuticle surface. This sequence suggests that the particles are formed and released from the cell surface, and then the particles become part of the external cuticle layer. The particles differ in size from those in the cytoplasm of the adjacent cell so may be formed at the cell membrane by enzyme action on precursor particles within the cell. ER, endoplasmic reticulum. Scale, 1 μm.

Figure 14 shows part of a cell process at the tip of outward extending hypodermal cells (Figure 4) from the base of a genital operculum. The cell membrane at this location is slightly retracted from the inner surface of the cuticle, and the likely stages of cuticle formation are evident. Small particles can be seen 1) at the cell membrane, 2) in the narrow space between the cell membrane and the inner cuticle surface and 3) at the inner surface of the cuticle. These membrane particles differ in size from those in the nearby cytoplasm.

**Figure 15:**
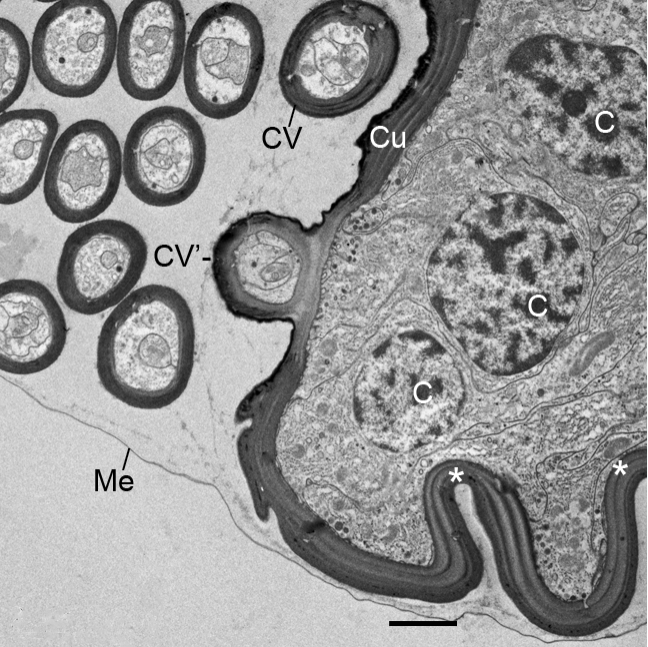
**Cluster of cuticular vesicles (CV) at the tip of the genital operculum after the third embryonic molt (stage 20)**. *Limulus polyphemus*, prone. TEM. The cuticular wall of these vesicles encloses plasma membrane and particles like that of nearby hypodermal cells (C), suggesting the vesicles are released from these cells at the appendage tip. One vesicle (CV') is attached to the cuticular tip as though it is in the process of release or uptake. Invaginations like those evident here (*) and a small cluster or cuticular vesicles are commonly seen at the tips of the genital operculum and first branchial appendage after the third molt (Figure 2A), but such vesicles were not seen in later stages. The vesicles appear to be held in place by a thin, transparent membrane (Me), probably the exuvium not yet removed from the appendages after the third molt. The cuticle (Cu) of the appendage tip is layered and much thicker than that of the vesicles as though they were released at an earlier time of development. Scale, 2 μm.

A small cluster of cuticular vesicles is commonly present at the tip of the genital operculum and first branchial appendage in the stage 20 interval between the third and fourth embryonic molts (Figure 2A). These vesicles are shown in more detail in the electron micrograph of Figure 15. The vesicles are held in place by a thin transparent membrane that covers part of the opisthosomal appendages at this stage. As explained below (Discussion), the membrane is probably exuvium. The cuticular vesicles can be seen dispersed away from the appendage tip if the membrane is disrupted as for the first branchial appendage in Figure 2A (asterisk).

Inside the cuticular vesicles there are plasma membranes and particles like those in the cytoplasm of nearby hypodermal cells (Figure 15). This suggests the cuticular vesicles are formed by extrusion (apocrine secretion) from these cells during or just after the third embryonic molt as the distal part of these appendages is extending from the opisthosomal ventrum (Figures 1B, 2A). In light and electron micrographs, the cuticular surface of the epithelial cells often has small invaginations and evaginations (e.g., asterisks in Figure 15) as though in the process of forming cuticular vesicles for eventual release.

### More advanced appendages, gill lamellae and space holders

As pointed out above, the embryos after their fourth molt are very active within their membranous covering, and the genital operculum and first branchial appendage beat rhythmically as much as 1-2/sec. During preparation of tissues for microscopy, the right and left genital operculum and branchial appendages were isolated with some overlying tissue, and the isolated structure continued beating erratically, starting and stopping and sometimes continuing at 1-2/sec. This indicates the presence of pacemaker nerve cells stimulating the bands of muscle cells in the appendages [1]. In adult horseshoe crabs, the isolated chain of opisthosomal ganglia produce rhythmic output patterns like those for ventilation and gill cleaning [40].

Compared with embryos after their third molt, the opisthosomal appendages and gill lamellae of the post fourth-molt embryos (stage 21) and first instars have thicker cuticle and smoother external surface with much less epithelial outgrowth along their length. The appendage and lamellar lumina often have fine traces of hemocyanin and an occassional hemocyte, an indication of the flow of hemolymph. In post fourth-molt embryos and beyond, there are more pillar-type trabeculae in appendages and gill lamellae, and these trabeculae are often at an advanced stage of development compared with embryos after their third molt. When examined with the light microscope, bands of muscle cells can be seen at the base of the anterior opisthosomal appendages [1].

**Figure 16:**
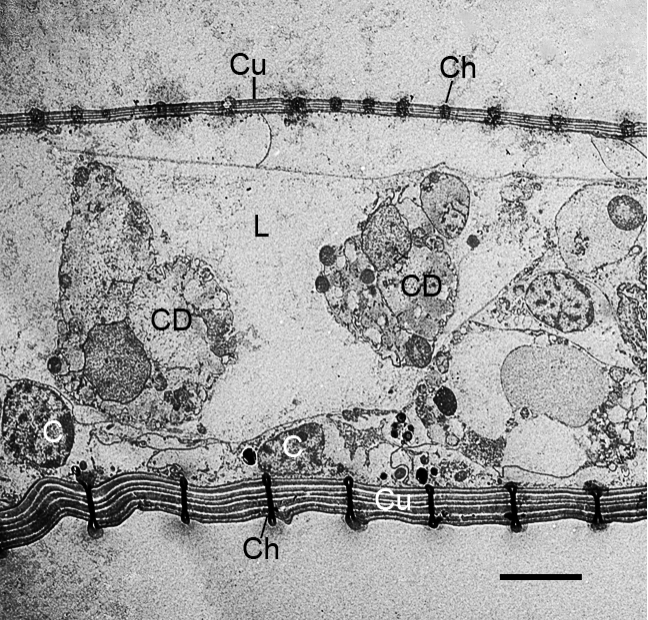
**Transverse section through the distal part of the genital operculum of a first instar**. *Limulus polyphemus*, prone. TEM. The external cuticle (Cu) is multi-layered, with the more protected dorsal cuticle (top) much thinner than that of the outer ventral surface (bottom). Some hypodermal cells (C) are present just inside the cuticle, and the lumen (L) has what appear to be clusters of cell debris (CD) from deteriorating cells, leaving space for the passage of hemolymph. In the developmental stages examined herein, the external cuticle of the genital operculum has gradually become thicker with regularly spaced channels (Ch) filled with an electron-opaque material. The fine particulate material just inside and outside these channels suggests some of the channel contents are released during prepartion of the tissues for microscopy. Scale, 5 μm.

**Figure 17:**
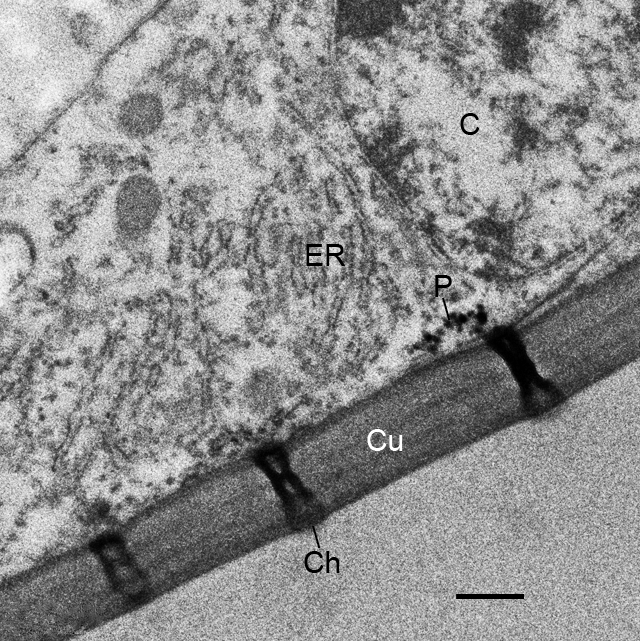
**Regularly spaced channels (Ch) in the external cuticle (Cu) of the genital operculum after the fourth molt (stage 21)**. *Limulus polyphemus*. TEM. The channels are partially or completely filled with electron-opaque material. The nearby hypodermal cell (C) has endoplasmic reticulum (ER) and small, dense particles (P) that may be the same material that eventually fills and is likely released from the external opening of the channels. Scale, 0.5 μm.

Sections through the genital operculum of post fourth-molt embryos and first instars show that the cuticle of the ventral surface is thicker than that of the dorsal, more protected surface (Figure 16). The cuticle now has layers, and there are regularly spaced channels (Figures 16, 17) usually all or partially filled with electron-opaque material that is probably secreted onto the cuticle surface.

In the gill lamellae of adult horseshoe crabs, the ventral surface of the central part of the gill has epithelial cells that appear to be specialized for ion transport [41]. In the present investigation, gill epithelial cells were not examined to determine if secretory and ion transport cells with specialized morphology are present in these early stages.

## Discussion

### Opisthosomal appendages and gill lamellae

The results herein are in agreement with earlier histological observations that the book gills arise as epithelial evaginations from the posterior surface of the branchial appendages [11,32]. Kingsley [11] also suggested there is some 'intucking' that might become more pronounced in producing the internal and anterior-directed air sacs of arachnid book lungs. In the present study there was no indication of epithelial invagination in the formation of individual gill lamella. Those authors [11,32] did not identify their tissue sections with a specific embryonic stage, since the stages 1-21 were characterized in later research [25,26].

From small bilateral ridges in the ventral surface of the opisthosoma (Figure 1A) [32], the distal part of the genital operculum and branchial appendages extend ventrally and become bilateral, immobile flaps in the third embryonic molt (Figure 1B) [1,32]. This clearly increases the surface area for gas exchange, and more striking changes continue in the interval (stage 20) between the third and fourth embryonic molts. The cellular outgrowth at the tip and along the length of the distal part of these appendages results in their knobby appearance at this stage (Figures 2A, B and 3, 4).

After the third embryonic molt (stage 20), a narrow cleft is evident between the bases of the opisthosomal appendages (Figures 2B, 3) as their proximal regions lengthen from the ventral surface of the opisthosoma. A thicker and distinctive proximal part of the appendages is formed. This basal region is filled with a diversity of cells and does not initially have a central lumen (Figures 3, 5, 8, 9). Some cells are deteriorating as though in the process of forming a lumen (Figures 3, 8). Other cells may migrate toward the periphery to become part of the hypodermal layer.

The gill lamellae begin as small cell clusters or an outward fold of hypodermis and cuticle at the posterior surface of the thick basal region of the first branchial appendage (Figures 1B, 5, 6, 7, 9, 12B) [32]. The development of the gill lamellae appears to be an elaboration of the general pattern of evaginating epithelial cell growth that is evident in the knobby appearance of the opisthosomal appendages in their early stages of formation (Figures 2A, B and 3, 4).

The basic structure of the gill lamellae is essentially the same as for the genital operculum and branchial appendages (Figures 2A, B and 3, 5, 6, 7, 8, 9), as though these structures are the result of a common program from the opisthosomal appendage anlage. That program includes the following: l) evagination and outgrowth of epithelium (Figures 5, 6, 9), 2) formation of a sac-like structure with central lumen (hemolymph channel) and pillar-type trabeculae (Figures 2A, B and 7, 9, 10, 11, 12A, B), 3) a hypodermal layer of epithelial cells (Figures 3, 7) and 4) typical apical-basal polarity of these epithelial cells, i.e., apical secretion of cuticle and the basal surface in contact with the hemolymph (Figures 3, 7, 14) [1,2,42,43].

### Endoskeleton

While the gill lamellae and distal parts of the opisthosomal appendages are ectodermal and epithelial (Figures 2A, B and 7, 9, 10, 11), the thick basal part of the opisthosomal appendages has mesodermal derivatives: hemocytes (Figures 6, 8), muscle (Figures 9, 12A) and endoskeleton (Figures 12A, B and 13). In their diagrams of embryo histological sections, Patten and Hazen [32] show that each opisthosomal segment has bilateral mesosomal somites, and the ventral cells of the somite are the source of the slender shaft of endoskeleton that eventually extends into the basal part of the genital operculum (Figures 12A, 13) and first branchial appendage (Figure 12B).

The endoskeleton has a thin electron-opaque matrix, and typically many matrix chambers have cell debris or cells with large vacuoles (Figure 13). This suggests the endoskeleton is deteriorating, but vacuolated cells are a common feature of invertebrate cartilage [37-39] including the cartilage of adult horseshoe crabs [35].

From observations herein, some endoskeleton is a common feature of the basal part of the genital operculum, present even in the first and second instars that were examined. Patten and Hazen [32] explained that their endoskeletal descriptions are based mainly on the genital operculum, and they assumed a similar pattern also occurs in the branchial appendages. They also note that the endoskeleton of the first branchial appendage is later in development than the endoskeleton of the genital operculum.

After the third and fourth embryonic molts in the present study, some endoskeleton is present at the base of the first branchial appendage in some specimens (Figure 12B), but in others there are large vacuoles that appear to be the remains of deteriorating cells (Figures 9, 12A). The endoskeleton may not yet have been formed in these latter examples, or possibly there is variable development of endoskeleton in these early stages.

From immobile flap-like structures after the third embryonic molt (Figures 1B, 2A, B), the appendages with gill lamellae become highly mobile after the fourth molt (Figures 7, 9, 12B) [1,32]. The somewhat variable endoskeletal conditions observed herein suggest there are changes in the endoskeleton associated with the onset of appendage mobility. This would occur especially in the endoskeleton of the genital operculum and first branchial appendage since these appendages have much development before their mobility begins after the fourth embryonic molt (stage 21).

Since the endoskeleton is produced from cells of the ventral somite [32], the proximal end of the endoskeletal shaft extends initially into the opisthosomal ventrum where the endoskeleton could impede appendage mobility. It is reasonable that some endoskeleton deteriorates or fails to form, especially at the proximal end (e.g., asterisk in Figure 12A), as the appendages become longer and mobile after the fourth embryonic molt. The longest lengths of endoskeleton were seen after the third embryonic molt (stage 20) while much smaller lengths were seen in later stages (Figure 12A, B). An endoskeleton has not yet been demonstrated at the base of the second and third branchial appendages that develop after the embryonic molts and hatching.

More detailed studies are needed to clarify the changing structure and role of the cartilage-like endoskeleton in these early stages. The evolutionary relationship of vertebrate and invertebrate cartilage is presently receiving much research attention [39]; also to be considered is the cartilage-like endoskeleton in the embryos and early instars of these 'living fossil' xiphosurids [44,45].

Endoskeletal tissue regarded as different types of cartilage is present in numerous invertebrates and at the base of opisthosomal appendages in adult horseshoe crabs [31,32,35-39]. The branchial endoskeletal matrix in adult horseshoe crabs has elastin, allowing support but also flexibility for the rhythmically moving appendages with attached gills [37]. The matrix that encloses the cells has protein fibrils in a metachromatic ground substance. The metachromatic staining property indicates the likely presence of the mucopolysaccharide, chondroitin sulfate [46]. This is further supported in biochemical studies [38,47,48].

### Cuticle

The pattern of cuticle formation evident in Figure 14 was commonly seen at the tip of outgrowing cell processes of the appendages and gill lamellae. Small, dense granules appear to be produced and released at the cell membrane, and these granules aggregate to form cuticle. Since the membrane particles differ in size from those inside the cell (Figure 14), there may be membrane enzymes that synthesize cuticle particles from precursor substances in the cell. A similar mode of cuticle formation is suggested in other arthropod developmental studies.

In an SEM investigation of book lung development in scorpion embryos, the cuticle of the air sac lamellae results from an aggregation of granular material released from the precursor cells [49]. Similar SEM observations are reported for cuticle formation for book gill development in the horseshoe crabs [1]. In a subsequent TEM study of scorpion book lungs, the initial cuticle of the air sac lamellae develops from particles formed at the plasma membrane of cell fragments released from the apical end of aligned precursor epithelial cells [2]. For spermatophore development in male scorpions, the cuticle is formed by joining small osmiophilic plates produced at the tips of microvilli of adjacent epithelial cells [50]. Some insect embryonic cuticle is apparently formed from small dense plaques produced by hypodermal cells with microvilli [51]. At the biochemical level in insects, a membrane enzyme (chitin synthase) polymerizes cytoplasmic precursor molecules and transfers the resulting chitin to the extracellular space [52].

In post-third molt embryos, cuticular vesicles are commonly seen at the tips of the flap-like genital operculum and first branchial appendage (Figures 2A, 15). The thin, transparent membrane that holds the vesicles in place is probably exuvium still attached to these appendages after the third molt. The exuvium from the embryonic molts remains inside the external embryonic membrane [1,26] and may be only partially removed from the opisthosomal appendages, especially in the early stages when appendages are not yet active.

No vesicles were seen at the tip of the second branchial appendage (Figure 2A, B), but this structure is relatively small in post-third molt embryos and may have been formed in the third molt (Figures 1B, 2A, B). After the third embryonic molt, numerous cuticular invaginations and evaginations are common at the tips of the genital operculum (asterisks in Figure 15) and first branchial appendage, as though these are sites of substantial growth. In Figure 15, the cuticle of the released vesicles is thinner than that of the nearby appendage tip, suggesting the cuticular vesicles are released earlier, i.e., during or just after the third embryonic molt. The significance and developmental role of these vesicles could not be determined from the light and electron micrographs examined herein.

### Living fossils

The oldest fossil horseshoe crab is from the Lower Ordovician [44], and in their review of the fossil record, Rudkin and Young [45] ask the question why the body plan of the horseshoe crab has been so persistent while others have not. From observing first and second instars in the laboratory, it appears the book gills are easily clogged with debris and ill-suited for long-term survival of the body plan.

The cuticle of the opisthosomal appendages of first instars has channels that probably release a protective substance (Figures 16, 17). An exocuticle, layered endocuticle and pore canals are present in the cuticle of adult horseshoe crabs [33]. The outer surface of their carapace is protected by an anti-fouling agent apparently secreted through pores in the cuticle [53]. Specialized epithelial cells at the base of transcuticular pores are described as the likely source of a slimy covering especially present in younger horseshoe crabs [31,54]. Gill cleaning behavior [55] and numerous moltings [26,56] may also help prevent clogging and infection of the page-like gills. Leibovitz and Lewbart [57] provide an informative review of the infections of the gills and other parts of horseshoe crabs.

Scorpions are another group with a 'relict' body plan. The oldest fossil from the Silurian period [45,58] looks much like modern forms. As reviewed by Dunlop [58], there has been much discussion about the phylogenetic relationship of scorpions and other chelicerates. Of course, the developmental sequence of animals is not a reliable indicator of their evolutionary history, but scorpion development should be considered since they have a conserved body plan. Pectines are commonly recognized as a distinctive feature of scorpions [58,59]. The pectines begin to develop very early in the embryo, suggesting an early separation of a clade with these structures. The indicators of terrestrialization, spiracles and book lungs, appear much later in the embryo after the pectines are prominent [59].

The late appearance of the book lungs in scorpion embryos can be compared with the late appearance of gill lamellae in the developmental sequence of structures in the opisthosoma of horseshoe crabs. For the latter, the results herein show the following sequence: 1) bilateral ridges in each opisthosomal segment (Figure 1A), 2) thin distal lobes of the opisthosomal appendages (Figures 1B, 2A), 3) thick proximal base of the opisthosomal appendages (Figure 2B), 4) eventual formation of gill lamellae, muscle bands and endoskeleton (Figures 5, 6, 7, 9, 12A, B and 13) [1] and 5) rhythmic appendage movement with swimming and aeration of the gills. The ability to add increasing numbers of gill lamellae as the animal increases in size, and the ability to use the opisthosomal appendages for swimming and for passage of water over the gills have surely been major factors for their survival.

### Comparison of development of book gills and scorpion book lungs

Muscle cells are not present within the scorpion book lungs, but there is some rhythmic agitation [60] produced by a process completely different from movement of book gills. Hypocardial ligaments extend from the ventral surface of the scorpion heart to the dorsal surface of the book lung sinus so that rhythmic heart contractions cause rhythmic movement of the book lungs. The hypocardial ligaments also contain muscle fibers which may be stretch-activated since no nerve fibers are evident. The book lungs, of course, have no endoskeleton.

In the formation of book gills, the primary result of precursor cell activity is the posterior-directed hemolymph sacs enclosed with cuticle and supported by pillar-type space holders (Figures 5, 6, 7, 9, 12A, B) [1]. The water channels without space holders are a secondary result of the repeated parallel hemolymph sacs (Figures 9, 12B) [1]. The sac-like hemolymph lamellae are formed mainly by proliferation and evagination of precursor epithelial cells followed by secretion of cuticle and development of a central hemolymph lumen (Figures 5, 6, 7, 9, 12B).

In the formation of scorpion book lungs, the primary result of precursor cell activity is the formation of internal, anterior-directed air sacs enclosed with cuticle and supported by specialized and diverse types of trabeculae [1,2,34,49]. The air sacs are produced by inward migration (ingression) of precursor cells from the parent invaginated epithelium (atrial wall) rather than evagination from an appendage base as occurs for book gills [1,2]. The hemolymph channels with pillar-type trabeculae are a secondary result of the parallel air sacs with their distinctive space holders.

For the development of scorpion air sacs, there is initial formation of parallel rows of precursor cells, and these cell rows anterior to the spiracle and atrium are the basis for the page-like air sacs [2,49]. The aligned cells release (apocrine secretion) cell fragments from their apical surface. Since the precursor cells are in parallel rows, their secreted cell fragments are also in parallel rows. In each row, the cell fragments gradually fuse into linear air channels with cuticular walls and trabeculae. Development of the cuticle walls is started with the formation of particles at the plasma membrane of the aligned cell fragments. Many of the aligned precursor cells deteriorate, leaving space for fluid in the primordial hemolymph channels. Some of these cells survive and become pillar-type trabeculae and a hypodermal layer for replacement of the air sac cuticle in the molt [2,49].

In the sac-like lamellae of both book gills and book lungs, the hypodermal cells have typical epithelial cell polarity with cuticle produced from the apical surface while their basal surface is in contact with the hemolymph [2,42,43]. As is common in invertebrate tissues [42], no basement membrane is secreted between the hemolymph and basal surface of the hypodermal cells (Figures 3, 7, 8, 10, 11).

The repeated and parallel pattern of the lamellae in book gills and book lungs is evident among the very first lamellae produced in embryos of horseshoe crabs (Figures 5, 9, 12B) [1] and scorpions [2,49]. As pointed out above, the basis of the spacing between lamellae is clearly different for the two types of respiratory organs. In spider and scorpion embryos, the air sacs are produced at the apical surface of the precursor cells previously aligned in parallel rows. This results in an air sac between each double row of cells, as evident in earlier light and electron micrographs [2,14-18].

In the formation of book gill lamellae, there is no initial alignment of precursor cells into parallel rows nor lamellar separation of aligned cells into double rows [1]. Rather, the primordial hypodermal cells apparently proliferate and migrate outward from a small fold or cluster (Figures 5, 6, 9, 12B), producing the opposed epithelial and cuticular walls of the lamellae (Figure 7). This results in some variation in the distance between gill lamellae and a varying number of hypodermal cells in the space between the lamellae (Figures 9, 12B). Thus, many page-like lamellae are formed for both book lungs and book gills, but there are substantial differences in cell activity for lamellar spacing and development in the two types of respiratory organs.

## Conclusions

These ultrastructural studies extend earlier observations [1,2,49] that the development of scorpion book lungs includes some distinctive cell activities not present in the formation of book gills: the initial inward migration (ingression) of precursor cells from the atrial epithelial layer, alignment of precursor cells into parallel rows, the secretion of cell fragments by the aligned cells, the resulting spacing of lamellae in a regular pattern of double cell rows and the development of a diversity of air sac trabeculae in addition to the pillar-type trabeculae present in the hemolymph channels of both book gills and book lungs [1,2,33,34,49].

There are no reports of structures like book gills that extend from the posterior surface of limb buds in scorpion embryos, although the early pectines suggest a gill-like function [2,49,59]. Whatever the evolutionary history of horseshoe crabs and scorpions, there are numerous cell activities that occur in the development of both book gills and book lungs: 1) epithelial cell proliferation apparently from opisthosomal appendage anlage, 2) cell migration, 3) side-by-side alignment of precursor cells, 4) apical-basal cell polarity with apical secretion of cuticle and the basal surface in contact with hemolymph, 4) formation of sac-like structures for passage of hemolymph or air, and the sacs have a central lumen and an outer wall of hypodermal cells with apical secretion of cuticle, 5) formation of pillar type space holders and 6) repeated formation of lamellae in a book-like pattern, although the cellular basis for the repeated lamellae is clearly different for book gills and book lungs.

As reviewed by Farley [1], since the end of the nineteenth century there have been numerous speculations about the book gill/book lung relation, based mainly on light microscopic observations. The similarities and differences in book gill/book lung development reported here and earlier [1,2,49] can probably elicit a diversity of interpretations including support for the original hypothesis that the similarities are indicative of common inheritance [6-15,18-21] while the differences in book gill/book lung development are adaptations for gas exchange in the different environments, aqueous and terrestrial.

## Materials and methods

A mixture of fertilized eggs and embryos of *Limulus polyphemus *(L.) were obtained from Gulf Specimens Marine Laboratory (GSML; Panacea, FL), and fertilized eggs were obtained from Marine Biological Laboratory (MBL; Woods Hole, MA). The eggs and embryos were dispersed into glass bowls (300 ml) about half filled with artificial sea water (Oceanic Sea Salt, Oceanic Systems, Dallas, TX). Specimens from the two locations were maintained in separate containers. The lighting and temperature (23-25°C) were ambient. The sea water was changed each 1-2 days, and the cultures were examined under the dissecting microscope to remove debris and deteriorating eggs and embryos.

The time of fertilization was unknown, so external features rather than days post fertilization are used to identify the developmental stage as described by Scholl [24] and Sekiguchi et al., [25,26]. The rate of development herein appears to be similar to that reported by those authors. First instars began to appear 24 days after the GSML eggs and embryos arrived, and 11 days later there were some second instars. The fertilized eggs from MBL seemed to develop more slowly. First instars appeared 30 days after egg arrival in the lab, and the second instars began to appear 47 days later.

Since first instar (trilobite) larvae do not feed [1], no food was added to the bowls described above. Second instars were transferred to larger bowls (1 liter) with aeration and food items. Some second instars were examined in this study, but the results are not included since the earlier stages provide informative examples of developing opisthosomal appendages and book gills.

Using artificial sea water as a dissecting medium, fine scissors and forceps were used to rupture the external covering, release the embryo and remove parts for microscopy. Tissues were prepared for SEM as described earlier [1]. For LM and TEM, the tissues were fixed 1-6 hours in 2.5% glutaraldehyde in 0.1 M cacodylate buffer (2-4°C). The tissues were washed in cold buffer and postfixed (4-12 hours, 2-4°C) in 1% OsO4 in 0.1 M cacodylate buffer. Tissues were washed again in buffer, dehydrated in a graded series of ethanol concentrations and embedded in Spurr's [61] plastic, modified for a new replacement component [62]. Twelve-fifteen hours was allowed for infiltration in 100% plastic, but this was not always sufficient for permeation of the early stage tissues of this study. The external cuticle becomes thicker with development, and embryos and larvae of this species are well known to tolerate variations in temperature, salinity, oxygen and pollutants [28].

Semi-thin and ultrathin sections were cut on a RMC MT-X microtome (Boeckeler Instruments). The latter sections were collected on grids pretreated with parlodion, stained with alcoholic uranyl acetate and lead citrate [63] and examined at 120 kv with a FEI Technai 12 (formerly Philips) electron microscope. Semi-thin sections were stained with warming and a mixture of 0.5 g toluidine blue, 0.5 g sodium borate and 20 ml of methyl alcohol in 200 ml H_2_0.

## Competing interests

The author declares that he has no competing interests.
